# Risk Factors of Sports-Related Injury in School-Aged Children and Adolescents: A Retrospective Questionnaire Survey

**DOI:** 10.3390/ijerph19148662

**Published:** 2022-07-16

**Authors:** Ryosuke Shigematsu, Shuta Katoh, Koya Suzuki, Yoshio Nakata, Hiroyuki Sasai

**Affiliations:** 1 Faculty of Education, Mie University, Tsu 514-8507, Mie, Japan; ka.shu1.tch@gmail.com; 2 School of Health and Sport Sciences, Chukyo University, Toyota 470-0393, Aichi, Japan; 3 Department of Sports Science, Juntendo University, Inzai 270-1695, Chiba, Japan; ko-suzuki@juntendo.ac.jp; 4 Faculty of Health and Sport Sciences, University of Tsukuba, Tsukuba 305-8577, Ibaraki, Japan; nakata.yoshio.gn@u.tsukuba.ac.jp; 5 Research Team for Promoting Independence and Mental Health, Tokyo Metropolitan Institute of Gerontology, Itabashi 173-0015, Tokyo, Japan; sasai@tmig.or.jp

**Keywords:** Bukatsu, extracurricular activities, injury epidemiology, Japan, school sports

## Abstract

Studies conducting quantitative surveys in school-aged children and adolescents help identify sports-related risk factors for acute and overuse injuries are scarce. This study aimed to quantify the risk factors for sports-related injury in school-aged children and adolescents by school categories. University students (*n* = 484) retrospectively recalled their sports experiences and related injuries in a questionnaire according to the following school categories: lower elementary school (LE), upper elementary school (UE), junior high school (JH), and high school (H). Both sports-related acute and overuse injuries were recorded. After adjusting various covariates, weekly hours in sports were identified as a significant risk factor in LE and UE. The interaction of weekly hours in sports and sports specialization was significant in LE and UE, suggesting that early specialization would be a risk factor in lower school categories. In JH and H, female sex, high-level competition, and injury experienced in a previous school category were significantly related to sports-related injuries. In conclusion, weekly hours in sports, high-level competitions, previous injury experience, and sex were confirmed as risk factors in specific school categories. Most identified risk factors are modifiable, suggesting that sports-related injuries can be prevented in school-aged children and adolescents.

## 1. Introduction

Adolescents in Japan are more physically active than those in other countries [1]. This is due to extracurricular sports activities, called Bukatsu, in Japanese schools. Appropriate physical activity—including sports activities—improves adolescents’ fitness, which may protect them against injury [2]. However, a sports-related injury is affected by time spent in sports, which has been recognized in literature as a risk factor for injuries [3,4]. Young athletes, who were aged between 7 and 18 years and trained in weekly hours over their age, were significantly more likely to be injured [3].

Our previous retrospective survey has found that weekly hours in sports increase as students go to higher school categories [5]. For example, lower elementary school (LE) students spent four hours, on average, per week in sports activities; however, upper elementary school (UE), junior high (JH), and high school (H) students engaged in sports for 8, 15, and 15 h per week, respectively [5]. Sports-related injuries increase in higher schooling categories, especially in JH and H. The proportion of injuries during the high school period was 41% [5], comparable to the findings reported in a US study of 75,298 high school students among 10 sports items [6]. Furthermore, in the Finnish 15 to 16-year-old cohort, 38% of the 1,386 males and 49% of the 1066 females, who participated in moderate-to-vigorous physical activity for 2 h per week or longer, reported low back pain [7].

Sports specialization has been found to be another potential risk factor for sports-related injuries. For example, the guidance from the American Academy of Pediatrics reported that those who participate in a variety of sports have fewer injuries and play sports longer than those who specialize before puberty [8]. Our previous study further revealed a slight variation in the number of sports experiences, particularly in JH and H, which demonstrates that the age at which specialization is begun in a single sport is 13 years [5].

Other factors such as psychological stress from high competition in schools [9], sports-starting age [10], sex [11], and injury experience [12] may further alter the risk of sports-related injuries in children and adolescents. Unfortunately, there has not yet been a comprehensive survey to determine each contributing risk factor. Previous systematic reviews determined each risk factor for a sports-related injury; however, there have been no studies that have examined the effect of multiple factors. By quantifying the effect size for each factor, an effective prevention strategy for sports-related injuries can be established. Therefore, this study aimed to quantify the risk factors for sports-related injury in school-aged children and adolescents. We hypothesized that each risk factor significantly relates to sport-related injuries regardless of the magnitudes of the impact.

## 2. Materials and Methods

This cross-sectional questionnaire survey was conducted from May to July 2017, to gather retrospective recalls, adhering to the Declaration of Helsinki. The ethical research committee of the Faculty of Education, Mie University, approved the study protocol (No. 2017-7, 8 March 2018). Furthermore, the summary of the protocol was registered at the University hospital Medical Information Network (UMIN) Clinical Trials Registry (UMIN 000036748).

**Figure 1:**
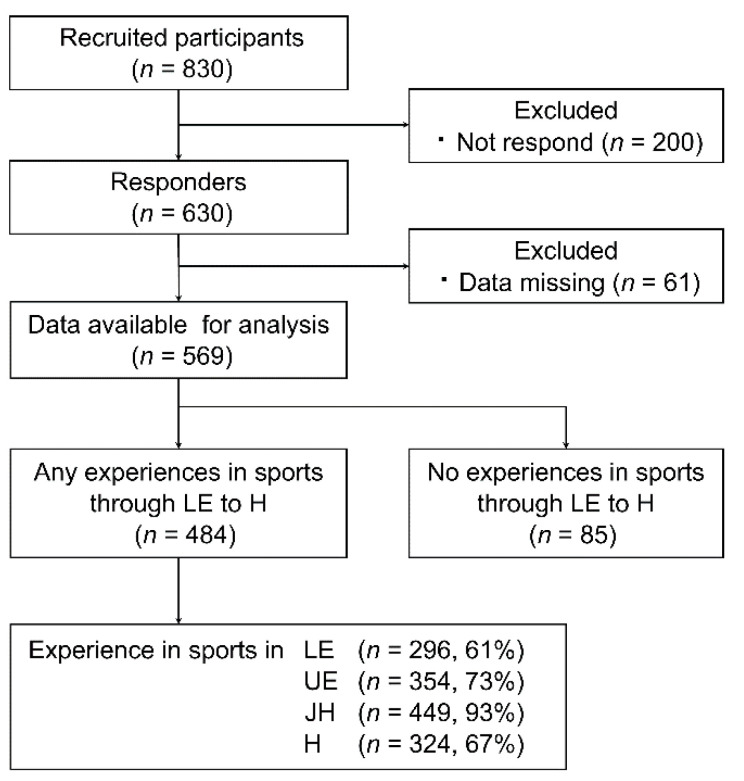
Flowchart of the selection of study participants. LE: lower elementary school, UE: upper elementary school; JH: junior high school; H: high school.

The study protocol has been detailed previously and can be accessed for more information [5]. In brief, a questionnaire was distributed to 830 students from a single university. The questionnaire collected data regarding the participants’ sports activities and sports-related injuries from LE to H (Appendix A). The data were divided into four categories according to school terms, namely LE, UE, JH, and H. Of the 570 valid responders, 484 had at least one sports experience through LE to H and served as study participants (Figure 1).

### 2.1. Term Definitions and Questionnaire

“Sports experiences” were defined as activities conducted at any sports organization, such as sports clubs and extracurricular activities (Bukatsu) at school. We excluded physical education classes in schools because all the participants attended these. Other recreational activities were also excluded because data for them were not documented. The term “sports item” denotes a sports event such as football, swimming, or dance. “Sports-related injuries” were defined as acute or overuse injuries [5] that occurred during or were caused by sports where a given participant could not participate in sports for one day or longer or were treated by a physician for two weeks or longer. Undiagnosed injuries were also included. Chronological tables were included in the questionnaire to attempt to minimize recall bias and enable participants to write their sports experiences and sports-related injuries in detail.

The questionnaire also asked the participants about their sex, sports-starting age, and the highest competition level, divided into national/prefectural or city competitions. Next, weekly hours in sports were calculated as a product of session frequency per week and hours per session. Data on weekly hours in each school category were then divided into four categories based on the 25th, 50th, and 75th percentiles.

### 2.2. Statistical Analysis

Fisher’s exact or chi-square test was utilized to compare proportions. Fisher’s exact test was also utilized as a post hoc test.

When determining each risk factor for sports-related injuries, a prevalence ratio (PR) with a 95% confidence interval (CI) was calculated using Poisson regression analysis with the assumption of constant over-dispersion. Poisson regression with robust variance could avoid problems of convergence, which have been described with log-binomial regression, especially when there are continuous independent variables [13].

All analyses were performed with jamovi v2.2.5.0 (Sydney, Australia) [14], built on top of the R statistical language. Statistical significance was set at 5%. Furthermore, when 95% CIs contain the value 1 and *p* values are less than 0.05, we focus on the *p* values rather than 95% CIs [15].

## 3. Results

### 3.1. Participants’ Characteristics, Sports Experience, and Sports-Related Injuries

The participants’ chronological ages in the data collection period ranged from 18 to 24 years (mean ± standard deviation: 20.1 ± 1.3 years). Their sports-starting ages ranged from 5 to 16 years (8.3 ± 2.9 years).

The proportion of those with one sports item was significantly higher than the proportion of those with multiple sports items in each school category (*p* < 0.01) (Table 1). Furthermore, only 10.0% and 0.9% of the students experienced multiple sports items during JH and H, respectively. The proportions of overall (acute and overuse) sports-related injuries significantly increased as school categories ascended, i.e., sports-related injuries in LE (4.7%) < UE (22.3%) < JH (33.9%) < H (39.5%) (Table 2).

### 3.2. Relationships between Sports-Related Injuries and Weekly Hours in Sports or Sports Specialization

Since weekly hours in sports were not normally distributed, the data were divided into quartiles in each school category. The numbers of sports-related injuries were then counted among the participants in each quartile in each school category (Table 3). The Chi-square test revealed that the proportions of sports-related injuries (acute, overuse, and overall) were significantly different according to weekly hours in sports in UE. In contrast, significant differences were not found in the other school categories.

**Figure 2:**
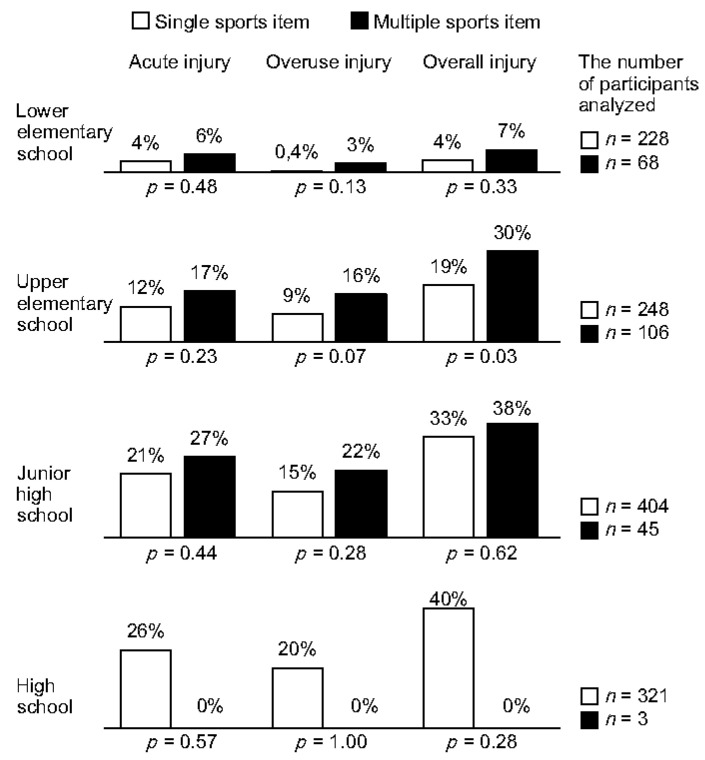
Proportions of those with sports-related injuries compared with single and multiple sports items. Overall injuries included acute and overuse injuries.

Figure 2 illustrates the proportions of those with sports-related injuries between those with single and multiple sports items. Those with multiple items were frequently injured in LE, UE, and JH. However, a significant difference was found in overall injuries in UE, suggesting that there is no significant association between sports specialization and sports-related injuries in many school categories.

To examine the associations between sports-related injuries and the weekly hours in sports and multiple sports items, both factors and the interaction between both factors were inputted, and adjusted PRs and 95% CIs were then calculated (Table 4). It was found that weekly hours in sports were positively associated with acute and overuse injuries in LE and UE (*p* < 0.05). Furthermore, multiple sports items were also positively associated with some types of injuries in LE and UE. The interactions of the two factors (specialization and weekly hours in sports) were significantly associated with overuse and overall injuries in UE. In JH and H, neither factors nor the interactions were significantly associated with any injuries.

When including other potential factors of injuries, such as sex, sports-starting age, and competition level into the models, it was found that weekly hours, multiple sports items, and their interaction were partially associated with some injuries in LE and UE; however, they were not associated with injuries in JH and H (Table 5). Being female was significantly related to acute injury in JH. On the other hand, it was a significant factor in low ratios of overuse in UE and JH and of acute and overall injuries in H. Sports experience in a national or prefectural competition was another significant risk factor for injuries in JH and H. Moreover, getting injured in a previous school category was found to be a significant risk factor of overuse and overall injuries in H.

## 4. Discussion

The novelty of the study is that it quantifies the effect size of multiple factors relating to sports-related injury. The most prominent finding of this study is that neither time spent playing sports nor sports specialization could completely explain the sports-related injuries through LE to H; nor could other factors.

Previous study findings have suggested that participation in extracurricular activities should be encouraged in school-aged children and adolescents because the activities are associated with higher levels of life satisfaction and optimism, and lower anxiety and depressive symptoms in students [16]. As these extracurricular activities include sport, it follows that an effective prevention strategy for sports-related injuries should be introduced to specific school categories.

### 4.1. Weekly Hours in Sports

Previous studies have reported a dose–response relationship between weekly hours in sports and sports-related injury [3,4]. The results of these studies are comparable to our results, but only with respect to UE. In UE, students who trained for less than 3 h per week (Q1) were less likely to get injured than those with 3 or more hours of training (Q2–Q4). Those with 3–7 h (Q2) were also less likely to get injured than those with 12 h or more (Q4). The number 12 in weekly hours is almost equivalent to the age of children attending UE; this result supports another study which shows that athletes aged 7–18 years, who participate in more weekly hours in sport than the number of their age, face increased odds of suffering a sports-related injury (odds ratio 1.59, 95% CI 1.17–2.16) [3].

In this study, we focused on weekly hours in sports because the Japan Sports Agency’s guidelines [17] recommend limiting weekly hours in sports to 11 h for JH and H students. This maximum limit was calculated as the sum of 2 h on four weekdays and 3 h on a weekend day. However, we could not confirm that the weekly hours in sports are a risk factor in the school categories in this study (Table 5). Note that the study participants experienced sports activities before the guidelines were enacted; therefore, they did not break the guidelines at any stage.

### 4.2. Specialization

This study found that most participants did not experience multiple sports items, particularly in JH (10.0%) and H (0.9%). This finding is in contrast with other previous studies showing that 71% of local high school students in the United States [18] and 61% of Finnish students aged 15–16 years [7] experienced multiple sports items. This difference could stem from the Japanese extracurricular activity (Bukatsu) system that was introduced in schools some decades ago. The Bukatsu system offers different kinds of sports or cultural clubs to students, and schools encourage students to belong to a single club, leading to sports specialization.

The analyses in this study do not indicate that sports specialization increases a risk of sports-related injuries in JH and H. Possible explanations for this are the slight variation in the number of sports items in these school categories and the sample size of participants with multiple sports items that are not enough to detect significant risk. Nevertheless, we recommend that students experience multiple sports items particularly during LE and UE because the interactions of sports specialization and sport time were significant, suggesting that injuries may decrease if students participate in multiple sports items in their given weekly hours in sports. This recommendation could be emphasized not only for injury prevention but also for an active lifestyle in their future [19]. In a study by Gallant et al. [19], late sports specialization in childhood was associated with a higher likelihood of recreational participation (relative risk 1.55, 95% CI 1.18–2.03) and a lower likelihood of nonparticipation (0.69, 0.51–0.93) in adolescence, compared with early sports specialization. The study recommends a late specialization in childhood.

### 4.3. Other Examined Risk Factors

We found that participation in either a national or prefectural sports competition was a significant risk factor for sports-related injuries in JH and H. This factor consists of two injuries: injury in competitions and injury during training. If an athlete or a team is at a high-performance level, they participate in many competitions throughout the year that require mechanical loads with ground surfaces or opponents. Furthermore, they are also expected to play at their highest abilities. This leads to competition-oriented injuries. For example, at least 11% of the athletes in the London Olympic games incurred an injury during the event [20]. Furthermore, their training exercise’s intensity and the fatigue that follows, which are likely to be high, also increases their risks of injuries. Psychological stress from competing in a regular position or the expectation of perfect performances can further increase the risk of sports-related injuries for athletes and possibly children and adolescents participating in national or prefectural sports competitions [9].

We also found that having a past experience of a sports injury in JH is a risk factor for sports-related injuries in H. This finding supports a previous study’s findings [12]. The mechanism of this association is unclear; however, injuries could often occur because injured body parts, regardless of their healing stage, have weak muscles, ligaments, and tendons that can affect an individual’s ability for adequate sports movement.

Contrary to our expectation, the sports-starting age was not detected as a risk factor for sports-related injuries. However, a previous study has outlined that if individuals start sports activities earlier, the mechanical exercise load on their specific body parts would be accumulated throughout their lives, leading to a high risk of injuries [21].

Being male was found to be a risk factor of overuse injury in UE and JH and acute and overall injury in H; however, being female was a risk factor of acute injury in JH. The inconsistent findings of this study are in contrast with those of previous studies, which have consistently reported that being female is a risk factor for acute, overuse, and overall injuries [11,21,22]. While the previous studies analyzed specific sports items, such as badminton and soccer, we did not separate the sports items in the current study. As we have previously reported [5], differences in sports items between men and women were significant in sports such as rubber-ball, baseball (men 40%, women 1%), and volleyball (8% and 18%, respectively). Consequently, rather than just sex, an interaction of sex with other covariates could explain the current results. For example, many female participants in this study started their sports activities in JH (19%-point increases compared to UE). Female students, particularly those at a beginner level, may participate in sports activities with seniors who are at a higher level and play in such an intensive manner that they exceed their abilities. This reason might explain the association of acute sports injuries with being female.

### 4.4. Limitations, Strengths, and Generalizability

Recalling previous sports-related injuries may have biased the study findings because the participants may not have remembered minor injuries they suffered as long as 10 years ago. This concern leads to underestimations of the magnitudes of the risk factors. In contrast, we believe that the possible risk factors, such as the weekly hours in sports, were precisely documented. Sports session frequency and hours per session varied in each sport item but were usually stable in each school. Therefore, the impact of the recall biases on our findings may not necessarily be substantial. Another limitation is the mixture of sports items in the analyses as more detailed results might have been obtained when considering different sport types. Furthermore, because the incidence and mechanisms of injury and the volume of exercise load vary dramatically among sports items [23], the risk factors examined in the study are likely to be over- or under-estimated.

One of this study’s strengths is that our results were derived from 12-year sports experiences, which can be applied to school education because the participants in the study were university students who graduated from schools and had not been elite athletes. If schools adequately adopt the research findings, injury occurrence and medical costs could be decreased. An effective strategy for sports injury prevention also contributes to athletes’ health and future sports activity; this can be done by making and improving policies on sports injury prevention. In a study conducted in Canada, a policy change disallowing body checking resulted in a 50% reduction in the injury rate and a 64% reduction in the concussion rate in 11–12-year-old hockey players [24]. If the Japanese Bukatsu guidelines [17] were improved according to the study findings, sports-related injuries in school children and adolescents would decrease. It has been found that students have been waiting for more effective strategies as 93% believe sports injuries could be prevented [25]. Therefore, efforts should be made to meet their expectations.

Further research is required to determine which components of high-level competitions are more definitively related to injury risks in JH and H. Furthermore, based on the results that sex was not interpretable in this study, future studies should investigate this factor.

## 5. Conclusions

To prevent sports-related injuries, we should pay attention to not only a single factor but also multiple factors such as weekly hours in sports, high-level competitions, previous injury experience, and sex, as they are partially confirmed as risk factors of sports-related injuries in specific school categories. However, sport specialization has not been confirmed in many schools. As most identified risk factors in this study are modifiable, this study suggests that sports-related injuries can be prevented in school-aged children and adolescents. In order to prevent injuries, we could practically advise (1) LE students to participate in multiple sports items in a certain sports time, (2) UE students to follow the same recommendation in LE and reduce sports time, particularly to reduce male students’ overuse injuries, (3) JH students to pay attention to overall injuries if they participate in high level competitions, and if they are female for acute injury and male for overuse injury, and (4) H students to pay attention to overuse and overall injuries if they participate in high level competitions, or if they have experienced injury in JH, and if they are male for acute and overall injuries.

## Figures and Tables

**Table 1:** The number of sports items for the study participants (*n* = 484).

School Category ^(1)^	Participants with Sports Experience	One *n* (%)	Multiple ^(2)^ *n* (%)	Two *n* (%)	Three *n* (%)	Four *n* (%)	*p* Value ^(3)^
LE	296	228 (77.0)	68 (23.0)	59 (19.9)	7 (2.4)	2 (0.7)	<0.01
UE	354	248 (70.1)	106 (29.9)	86 (24.3)	16 (4.5)	4 (1.1)	<0.01
JH	449	404 (90.0)	45 (10.0)	42 (9.4)	3 (0.7)		<0.01
H	324	321 (99.1)	3 (0.9)	3 (0.9)			<0.01

^(1)^ LE, lower elementary school; UE, upper elementary school; JH, junior high school; and H, high school. ^(2)^ Multiple sports item proportion: H (0.9%) < JH (10.0%) < LE (23.0%) < UE (29.9%). ^(3)^ Proportion tests between one and multiple sports items.

**Table 2:** The number of sports-related injuries in the study participants (*n* = 484).

Variable	School Category ^(1)^	Participants with Sports Experience	None *n* (%)	One Injury or More *n* (%)	*p* Value ^(2)^
Acute	LE	296	284 (95.9)	12 (4.1)	<0.01
injuries	UE	354	307 (86.7)	47 (13.3)	<0.01
	JH	449	352 (78.4)	97 (21.6)	<0.01
	H	324	241 (74.4)	83 (25.6)	<0.01
	Injury proportion: LE (4.1%) < UE (13.3%) < JH (21.6%), H (25.6%)				
Overuse	LE	296	293 (99.0)	3 (1.0)	<0.01
injuries	UE	354	314 (88.7)	40 (11.3)	<0.01
	JH	449	377 (84.0)	72 (16.0)	<0.01
	H	324	260 (80.2)	64 (19.8)	<0.01
	Injury proportion: LE (1.0%) < UE (11.3%) < JH (16.0%) < H (19.8%)				
Overall	LE	296	282 (95.3)	14 (4.7)	<0.01
injuries ^(3)^	UE	354	275 (77.7)	79 (22.3)	<0.01
	JH	449	297 (66.1)	152 (33.9)	<0.01
	H	324	196 (60.5)	128 (39.5)	<0.01
	Injury proportion: LE (4.7%) < UE (22.3%) < JH (33.9%) < H (39.5%)				

^(1)^ LE, lower elementary school; UE, upper elementary school; JH, junior high school; and H, high school. ^(2)^ Proportion tests between none and one injury or more. ^(3)^ Overall injuries included acute and overuse injuries.

**Table 3:** The number and proportion of participants with sports-related injuries according to weekly hours in sports.

Weekly Hours in Sports ^(1)^			*n*	Acute Injury		Overuse Injury		Overall Injury	
				None *n* (%)	Any *n* (%)	None *n* (%)	Any *n* (%)	None *n* (%)	Any *n* (%)
Lower elementary school									
	Q1	1 ≦ x ˂ 2	30	29 (97)	1 (3)	30 (100)	0 (0)	29 (97)	1 (3)
	Q2	2 ≦ x ˂ 4	109	107 (98)	2 (2)	109 (100)	0 (0)	107 (98)	2 (2)
	Q3	4 ≦ x ˂ 8	68	65 (96)	3 (4)	67 (99)	1 (1)	64 (94)	4 (6)
	Q4	8 ≦ x ≦ 35	89	83 (93)	6 (7)	87 (98)	2 (2)	82 (92)	7 (8)
	Total		296		*p* = 0.38		*p* = 0.41		*p* = 0.23
Upper elementary school									
	Q1	1 ≦ x ˂ 3	75	72 (96)	3 (4)	73 (97)	2 (3)	70 (93)	5 (7)
	Q2	3 ≦ x ˂ 7	94	83 (88)	11 (12)	86 (91)	8 (9)	76 (81)	18 (19)
	Q3	7 ≦ x ˂ 12	84	71 (85)	13 (15)	74 (88)	10 (12)	65 (77)	19 (23)
	Q4	12 ≦ x ≦ 35	101	81 (80)	20 (20)	81 (80)	20 (20)	64 (63)	37 (37)
	Total		354		*p* = 0.02 ^(2)^		*p* = 0.01 ^(2)^		*p* = 0.01 ^(3)^
Junior High School									
	Q1	1 ≦ x ˂ 12	109	84 (77)	25 (23)	92 (84)	17 (16)	73 (67)	36 (33)
	Q2	12 ≦ x ˂ 16	135	107 (79)	28 (21)	109 (81)	26 (19)	86 (64)	49 (36)
	Q3	16 ≦ x ˂ 20	72	57 (79)	15 (21)	63 (88)	9 (12)	50 (69)	22 (31)
	Q4	20 ≦ x ≦ 42	133	104 (78)	29 (22)	113 (85)	20 (15)	88 (66)	45 (34)
	Total		449		*p* = 0.98		*p* = 0.61		*p* = 0.86
High School									
	Q1	1 ≦ x ˂ 12	80	60 (75)	20 (25)	67 (84)	13 (16)	52 (65)	28 (35)
	Q2	12 ≦ x ˂ 15	52	38 (74)	13 (26)	42 (82)	9 (18)	34 (65)	18 (35)
	Q3	15 ≦ x ˂ 20	91	70 (77)	21 (23)	73 (80)	18 (20)	56 (62)	35 (38)
	Q4	20 ≦ x ≦ 48	101	72 (71)	29 (29)	77 (76)	24 (24)	54 (53)	47 (47)
	Total		324		*p* = 0.84		*p* = 0.62		*p* = 0.36

^(1)^ Weekly hours in sports in each school category were divided into four categories by quantities. The lowest values in Q1 and the highest values in Q4 in each school category are the minimum and maximum weekly hours in sports, respectively. ^(2)^ Post hoc tests revealed that Q1 < Q4. **^(^**^3)^ Post hoc test revealed that Q1 < Q2, Q3, Q4, and Q2 < Q4.

**Table 4:** Adjusted prevalence ratio with 95% confidential intervals of sports-related injuries according to school categories using three covariates.

School Category	Injury	Covariate	PR (95% CI) ^(1)^	*p* Value ^(2)^
Lower elementary school	Acute	Weekly hours in sports (A)	2.11 (0.92–4.01)	0.04 *
		Multiple sports items (B)	1.07 (0.97–1.16)	0.14
		(A) × (B)	0.96 (0.82–1.11)	0.60
	Overuse	Weekly hours in sports (A)	4.78 (2.10–10.59)	<0.01 *
		Multiple sports items (B)	1.19 (1.07–1.33)	<0.01 *
		(A) × (B)	0.87 (0.73–0.99)	0.05
	Overall	Weekly hours in sports (A)	2.47 (1.23–4.39)	0.01 *
		Multiple sports items (B)	1.09 (1.00–1.17)	0.03 *
		(A) × (B)	0.94 (0.82–1.06)	0.35
Upper elementary school	Acute	Weekly hours in sports (A)	1.55 (1.04–2.19)	0.02 *
		Multiple sports items (B)	1.07 (1.02–1.11)	<0.01 *
		(A) × (B)	0.96 (0.90–1.02)	0.15
	Overuse	Weekly hours in sports (A)	1.55 (1.00–2.28)	0.04 *
		Multiple sports items (B)	1.08 (1.03–1.13)	<0.01 *
		(A) × (B)	0.93 (0.87–0.99)	0.03 *
	Overall	Weekly hours in sports (A)	1.55 (1.16–2.02)	<0.01 *
		Multiple sports items (B)	1.07 (1.04–1.11)	<0.01 *
		(A) × (B)	0.94 (0.90–0.99)	0.02 *
Junior high school	Acute	Weekly hours in sports (A)	1.16 (0.63–1.91)	0.61
		Multiple sports items (B)	1.00 (0.97–1.03)	0.83
		(A) × (B)	1.00 (0.92–1.09)	0.92
	Overuse	Weekly hours in sports (A)	1.50 (0.82–2.48)	0.15
		Multiple sports items (B)	0.99 (0.96–1.03)	0.61
		(A) × (B)	1.02 (0.93–1.10)	0.71
	Overall	Weekly hours in sports (A)	1.29 (0.85–1.87)	0.20
		Multiple sports items (B)	0.99 (0.97–1.02)	0.64
		(A) × (B)	1.01 (0.95–1.07)	0.75
High school ^(^^3)^	Acute	Weekly hours in sports	0.99 (0.97–1.02)	0.61
	Overuse	Weekly hours in sports	1.02 (0.99–1.05)	0.26
	Overall	Weekly hours in sports	1.00 (0.98–1.03)	0.71

^(1)^ PR: Prevalence ratio, CI: Confidence interval. ^(2)^ * *p* < 0.05. ^(3)^ Multiple sports items were not included in the high school category as only 3 of 324 participants experienced multiple sports items, resulting in an inability to calculate a 95% CI.

**Table 5:** Adjusted prevalence ratio with 95% confidential interval of sports-related injuries according to school categories using multiple covariates.

School Category	Injury	Covariate	PR (95% CI) ^(1)^	*p* Value ^(2)^
Lower elementary school	Acute	Female	1.35 (0.39–4.66)	0.63
		Sports-starting age (1 year late)	0.90 (0.48–1.74)	0.73
		National or prefectural competition	1.02 (0.88–1.18)	0.82
		Multiple sports items (A)	1.06 (0.90–1.22)	0.44
		Weekly hours in sports (B)	1.07 (0.97–1.16)	0.15
		(A) × (B)	0.99 (0.99–1.01)	0.45
	Overuse ^(3)^	Female	2.26 (0.67–8.79)	0.20
		Sports-starting age (1 year late)	0.67 (0.33–1.27)	0.24
		Multiple sports items (A)	1.53 (1.18–2.55)	0.02 *
		Weekly hours in sports (B)	1.28 (1.14–1.58)	<0.01 *
		(A) × (B)	0.97 (0.94–0.99)	0.01 *
	Overall	Female	1.48 (0.46–4.70)	0.50
		Sports-starting age (1 year late)	0.87 (0.48–1.61)	0.63
		National or prefectural competition	1.04 (0.91–1.20)	0.58
		Multiple sports items (A)	1.08 (0.93–1.24)	0.29
		Weekly hours in sports (B)	1.08 (0.99–1.17)	0.05
		(A) × (B)	0.99 (0.97–1.01)	0.25
Upper elementary school	Acute	Female	1.04 (0.55–1.95)	0.90
		Sports-starting age (1 year late)	1.14 (0.83–1.61)	0.42
		National or prefectural competition	1.02 (0.95–1.10)	0.55
		Injury experience ^(4)^	1.75 (0.56–4.30)	0.27
		Multiple sports items (A)	1.06 (0.98–1.14)	0.12
		Weekly hours in sports (B)	1.04 (0.99–1.09)	0.10
		(A) × (B)	0.99 (0.98–1.00)	0.04 *
	Overuse	Female	0.20 (0.06–0.53)	<0.01 *
		Sports-starting age (1 year late)	0.90 (0.64–1.27)	0.53
		National or prefectural competition	1.08 (0.99–1.20)	0.11
		Injury experience ^(4)^	1.47 (0.35–4.21)	0.53
		Multiple sports items (A)	1.08 (0.99–1.18)	0.10
		Weekly hours in sports (B)	1.06 (1.00–1.12)	0.04 *
		(A) × (B)	0.99 (0.97–1.00)	0.06
	Overall	Female	0.61 (0.37–0.99)	0.05
		Sports-starting age (1 year late)	0.99 (0.79–1.42)	0.92
		National or prefectural competition	1.04 (0.99–1.10)	0.15
		Injury experience ^(4)^	1.24 (0.50–2.60)	0.60
		Multiple sports items (A)	1.06 (1.01–1.12)	0.02 *
		Weekly hours in sports (B)	1.05 (1.01–1.09)	0.01 *
		(A) × (B)	0.99 (0.98–1.00)	0.01 *
Junior high School	Acute	Female	1.68 (1.09–2.58)	0.02 *
		Sports-starting age (1 year late)	1.04 (0.92–1.17)	0.54
		National or prefectural competition	1.08 (1.02–1.13)	0.01 *
		Injury experience ^(4)^	1.24 (0.75–1.98)	0.39
		Multiple sports items (A)	1.03 (0.96–1.09)	0.43
		Weekly hours in sports (B)	1.00 (0.97–1.03)	0.95
		(A) × (B)	1.00 (0.99–1.01)	0.92
	Overuse	Female	0.45 (0.224–0.80)	0.01 *
		Sports-starting age (1 year late)	1.12 (0.98–1.28)	0.10
		National or prefectural competition	1.06 (1.00–1.13)	0.04 *
		Injury experience ^(4)^	1.58 (0.93–2.64)	0.09
		Multiple sports items (A)	1.02 (0.95–1.10)	0.52
		Weekly hours in sports (B)	0.98 (0.94–1.01)	0.26
		(A) × (B)	1.00 (0.99–1.01)	0.79
	Overall	Female	1.08 (0.79–1.47)	0.62
		Sports-starting age (1 year late)	1.04 (0.95–1.13)	0.36
		National or prefectural competition	1.07 (1.04–1.12)	<0.01 *
		Injury experience ^(4)^	1.27 (0.91–1.76)	0.16
		Multiple sports items (A)	1.00 (0.95–1.05)	0.94
		Weekly hours in sports (B)	0.99 (0.97–1.02)	0.65
		(A) × (B)	1.00 (0.99–1.01)	0.98
High school ^(5)^	Acute	Female	0.55 (0.35–0.84)	0.01 *
		Sports-starting age (1 year late)	1.04 (0.97–1.11)	0.30
		National or prefectural competition	1.03 (0.98–1.08)	0.21
		Injury experience ^(4)^	1.03 (0.69–1.52)	0.87
		Weekly hours in sports	0.99 (0.97–1.02)	0.54
	Overuse	Female	1.04 (0.63–1.67)	0.88
		Sports-starting age (1 year late)	1.03 (0.94–1.12)	0.51
		National or prefectural competition	1.10 (1.04–1.19)	<0.01 *
		Injury experience ^(4)^	1.83 (1.16–2.92)	0.01 *
		Weekly hours in sports	1.02 (0.99–1.04)	0.27
	Overall	Female	0.72 (0.53–0.98)	0.04 *
		Sports-starting age (1 year late)	1.04 (0.98–1.09)	0.18
		National or prefectural competition	1.04 (1.01–1.08)	0.03 *
		Injury experience ^(4)^	1.34 (1.00–1.77)	0.04 *
		Weekly hours in sports	1.01 (0.99–1.02)	0.50

^(1)^ PR: Prevalence ratio, CI: Confidence interval. ^(2)^ * *p* < 0.05. ^(3)^ Excluded “national or prefectural competition” because only three participants in the competition got overuse injury. ^(4)^ Injury experiences (either acute or overuse) in the previous school category. ^(5)^ Multiple sports items were not included in the high school because only 3 of 324 participants experienced multiple sports items, resulting in an inability to calculate a 95% CI.

## Data Availability

The data are not publicly available in compliance with the investigation confidentiality.

## Appendix A. Questionnaire

1. Have you ever participated in any organized sports activities, such as extracurricular activities (Bukatsu) or sports club activities? If yes, please proceed to the next question. If no, please stop responding to the questionnaire and hand it to the research staff.

<Yes/No>

2. Please fill in information in the table regarding your sports experiences from elementary school to high school. You may refer to the example below. The term “sport experiences” is defined as any activity in sports organizations such as Bukatsu and sports clubs. However, the term excludes physical education classes in schools and recreational activities. The term “sports-related injuries” is defined as acute injuries or overuse injuries, which occurred during or due to sports activities, such that you could not participate in any sports activities for a day or longer or you were treated by a physician for two weeks or longer.

Example

**Sports Experience**	**Age**	**Sports-Related Injuries**
Swimming	9	
Swimming	10	
Basketball	11	Jumper's knee
Athletics	12	Jumper's knee, back pain
Athletics	13	Jumper's knee, back pain

**Sports Experience**	**Age**	**Sports-Related Injuries**
	7	
	8	
	9	
	10	
	11	
	12	
	13	
	14	
	15	
	16	
	17	
	18	

3. Please describe in detail your sports experiences. Similar to the example below, please use one column per experience.

(1) If you participated in seasonal sports such as skiing for multiple years, fill in information for the entire month. For example, if you participated in an activity for 2 months for 2 years, the total number of months is four.

(2) In case you require more columns, please use the backside of this page and add columns.

Example

4. Please describe in detail your sports-related injuries. Similar to the example below, please use one column for one injury.

(1) If you do not remember the proper diagnosis, please describe the injury in as much detail as possible.

(2) In the bracket for duration, please fill the number of months you required for complete recovery.

(3) If an injury has not been treated yet, please count the length of duration from the month of injury to the month of high school graduation.

Example

5. Please fill in your characteristics below.

(1) Sex:

(2) Age (yr):

(3) Height (cm):

(4) Weight (kg):

(5) Sports start age (yr):
